# Ciprofloxacin Alone vs. Ciprofloxacin plus an Aminoglycoside for the Prevention of Infectious Complications following a Transrectal Ultrasound-Guided Prostate Biopsy: A Retrospective Cohort Study

**DOI:** 10.3390/antibiotics12010056

**Published:** 2022-12-29

**Authors:** Daniel J. G. Thirion, Jean-Alexandre Caissy, Florence Poulin, Camille S. H. Lanfranchi, Albin Deda, Armen Aprikian, Charles Frenette, Sero Andonian

**Affiliations:** 1 Faculty of Pharmacy, Université de Montréal, Montreal, QC H3C 3J7, Canada; 2 Pharmacy Department, Royal Victoria Hospital, McGill University Health Centre, Montreal, QC H4A 3J1, Canada; 3 Faculty of Sciences, University of Geneva, 1211 Geneva, Switzerland; 4 Department of Surgery, Royal Victoria Hospital, McGill University Health Centre, Montreal, QC H4A 3J1, Canada; 5 Infectious Diseases Department, Royal Victoria Hospital, McGill University Health Centre, Montreal, QC H4A 3J1, Canada; 6 Urology Department, Royal Victoria Hospital, McGill University Health Centre, Montreal, QC H4A 3J1, Canada

**Keywords:** infection control, antimicrobial resistance, antibiotic prophylaxis, augmented prophylaxis, multidrug resistance, targeted prophylaxis, antimicrobial stewardship

## Abstract

The purpose of this study was to evaluate the impact of augmented prophylaxis (ciprofloxacin augmented with an aminoglycoside) compared with that of empirical prophylaxis (ciprofloxacin alone) on transrectal post-prostate biopsy infectious complication (PBIC) rates. A retrospective cohort study evaluated 2835 patients receiving either augmented or empirical prophylactic regimen before undergoing a transrectal ultrasound-guided prostate biopsy between January 2010 and October 2018. The patients were compared according to prophylactic regimen received. The incidence of PBICs and the impact of risk factors were evaluated. A total of 1849 patients received the empirical regimen, and 986 patients received the augmented regimen. The composite PBIC rate was 2.1% (*n* = 39) and 0.9% (*n* = 9) (*p* = 0.019), respectively, and the SIRS rate was 1.9% and 0.8% (*p* = 0.020), respectively. Of the 50 patients presenting with a PBIC, 29 (58%) had positive cultures (blood and/or urine) for *Escherichia coli*, of which 28 (97%) were ciprofloxacin-resistant. Taking a fluoroquinolone in the previous 6 months and having a previous urinary tract infection within 1 year prior to the biopsy had significant impact on PBIC rates (*p* = 0.009 and *p* = 0.011, respectively). Compared with ciprofloxacin alone, augmented prophylaxis was associated with significantly lower PBICs.

## 1. Introduction

The rate of transrectal post-prostate biopsy infectious complications (PBICs) has been increasing rapidly worldwide in recent years [1,2,3]. During a transrectal ultrasound-guided prostate biopsy (TRUSPB), microorganisms from the rectal flora can gain access to the sampling site through the needle used for the biopsy. Antibiotic prophylaxis has been shown to be the most effective approach for decreasing infectious complication rates for this procedure [4].

In parallel with the increase in infectious complications in recent years, an increase in the prevalence of multidrug-resistant microorganisms has been observed [2]. Fluoroquinolone-resistant (FQ-R) microorganisms are associated with the PBICs’ increase, particularly *Escherichia coli*, as it is the most common pathogen associated with PBICs [2,5,6,7,8]. In addition to FQ-R *E. coli*, strains of trimethoprim-sulfamethoxazole-resistant *E. coli* and extended spectrum beta-lactamase-producing *E. coli* have been found in cultures of patients experiencing PBICs [8]. Resistance patterns vary widely depending on geographic regions, although fluoroquinolone resistance is widespread throughout the world [2,7,9]. When antibiotics are given as a prophylactic measure, patients colonized with resistant microorganisms are more likely to be affected by infectious complications and hospitalizations post-prostate biopsy than patients who are not (by four-fold) [10]. With the spread of antibiotic resistance, antibiotic prophylaxis is shown to be ineffective in more and more cases [2].

Choosing an optimal prophylactic regimen is based on risk factors that are classified in procedure-specific characteristics and on patient characteristics [11,12]. Procedure-specific characteristics include the choice of approach, which can be transrectal or perineal, and the sterilization conditions, for instance. For a transrectal ultrasound-guided prostate biopsy (TRUSPB) procedure, contamination of the needle should be considered, and the chosen prophylactic antibiotics should cover the pathogens of the gastrointestinal flora [11]. Patient characteristics include, among others, age and colonization status [12]. Consideration of these characteristics and the implementation of corresponding strategies can help prevent infectious complications. When a prophylactic regimen is correctly adapted to pathogen and resistance patterns, antibiotic prophylaxis reduces PBICs rates [4].

The current guidelines suggest giving patients a single dose of an oral fluoroquinolone 60 min before the procedure as first-line antibiotic prophylaxis [11,13]. As of 2019, because of the risk for PBICs caused by the increasing prevalence of FQ-R microorganisms, fluoroquinolones are no longer indicated for prostate biopsy in European Union countries [9]. The increasing resistance is forcing clinicians to attempt other methods, such as targeted and augmented prophylaxis. The effectiveness of targeted prophylaxis is still debated [14,15,16,17,18]. Indeed, rectal flora sampling can identify patients at risk of complication; however, targeted prophylaxis does not significantly reduce the risk of complications in comparison with augmented prophylaxis [15,16,17,18]. This could be due to the heavy bacterial variability of rectal swabs that influence the clinical impact of targeted prophylaxis [19]. Augmented prophylaxis, based on the addition of another antibiotic to the current regimen, seems to be more effective than empirical and targeted prophylaxis in the prevention of PBICs [16,20]. This strategy consists of broadening the antibiotic spectrum to cover for possible resistance. However, the effectiveness of adding an AMG to a standard ciprofloxacin regimen in decreasing PBIC rates is controversial [20,21,22,23]. The aim of this study was to evaluate the impact of augmented prophylaxis (ciprofloxacin augmented with an aminoglycoside) compared with the impact of empirical prophylaxis (ciprofloxacin alone) on transrectal post-prostate biopsy infectious complication rates.

## 2. Materials and Methods

### 2.1. Design and Setting

This retrospective cohort study was conducted on all men 18 years old or older that underwent a TRUSPB at a tertiary care facility. Patients were included if they received either ciprofloxacin alone (Cipro group) or ciprofloxacin combined with an AMG (CiproAMG group) as prophylaxis between January 2010 to November 2018. This study was approved by the McGill University Health Centre (MUHC) Ethics Committee.

### 2.2. Antibiotic Prophylaxis

From January 2010 to November 2018, a 3-day regimen of ciprofloxacin (500 mg twice daily or 1 g once daily) was given, starting the day before the biopsy, without bowel preparation. Administration the day of surgery was done in clinic within 1 h of the procedure. As of 2011, select urologists started to augment this regimen with a single 80 mg dose of an AMG (gentamicin or tobramycin) given intramuscularly 30 to 60 min before the procedure. Gentamicin was initially used and was eventually switched to tobramycin, according to hospital formulary. Logistics favored IM administration of a fixed 80 mg dose. The timing of the administration remained constant throughout the study. All other infection control policies and procedures for this procedure remained unchanged during the study period.

### 2.3. Outcomes

PBICs were defined as systemic inflammatory response syndrome (SIRS), sepsis, septic shock, and bacteremia in symptomatic patients [24]. The identification of patients with complications was done through a chart review of all patients admitted to the MUHC emergency room within 30 days post-biopsy. PBICs were adjudicated by two experts, either urologists or infectious diseases specialists. In case of disagreement, a third expert made the decision.

### 2.4. Data Collection

Baseline demographic data (i.e., date of birth, age, weight, and hospital), biopsy data (i.e., date of biopsy and number of cores removed), and prophylaxis data (i.e., antibiotic choice, dose, timing, and duration and the appropriateness of prophylaxis) were collected. Data regarding risk factors included having a TRUSBP, a hospitalization or urinary tract infection (UTI) within the previous year, fluoroquinolone (FQ) use within the 6 previous months, and the number of cores. Outcomes, pathogens, and susceptibilities were collected for patients that developed a PBIC. Patients that developed a PBIC and that were administered to a different emergency site were referred to their caring surgeon at the MUHC, as per local regulations. In addition, an infection control nurse monitored the provincial database for the rare event in which a patient was admitted to another institution. All data was collected by manual review of the local electronic database.

### 2.5. Data Analysis

The study cohort was categorized into the Cipro or CiproAMG group; these two groups were compared using a Chi-square test or a Student’s t-test, as appropriate, with regards to demographic, biopsy, and risk factors variables. The PBICs were analyzed in relation to the treatment group using a Chi-square test (SPSS package version 25). Risk factors and demographic variables that significantly differed between treatment groups were first analyzed by univariate logistic regression analysis. Composite PBICs’ occurrence was the dependent variable. If the *p*-value was inferior to 0.1, a multivariate logistic regression analysis was used. A *p*-value below 0.1 was chosen, given the sparsity of patients presenting risk factors. The multivariate logistic regression allowed the minimization of bias when assessing the impact of the treatment on outcome. A linear regression was used to determine if a positive or negative trend in administered regimen through the years was significant. For all statistical comparisons, a difference between groups was found to be significant if the *p*-value was inferior to 0.05.

### 2.6. Sample Size Calculation

A sample size of 1175 to 2280 patients was required to determine a significant difference in infectious complication rates, using a two-sided alpha of 0.05 and a power of 80%. The PBIC rate was assumed to be 4–5% following ciprofloxacin prophylaxis alone, and 1.5–2% following the augmented regimen [22,25].

## 3. Results

**Figure 1:**
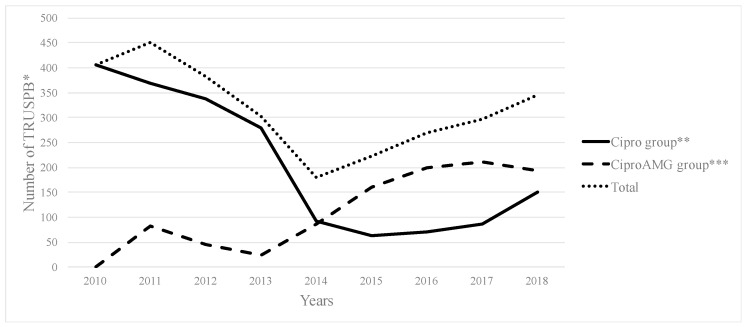
Number of transrectal ultrasound-guided prostate biopsies performed from 2010 to 2018. The number of transrectal ultrasound-guided prostate biopsies per year are shown in a linear trend and are stratified according to groups (Cipro group and CiproAMG group) and total number of biopsies performed. * Transrectal ultrasound-guided prostate biopsy. ** Significant negative trend. *** Significant positive trend.

A total of 4346 TRUSPBs were performed between January 2010 and November 2018. Of these, 1511 were excluded due to a lack of information (*n* = 937) or because of a prophylactic regimen that differed from that of the study (*n* = 574). In all, 2835 procedures were included, 1849 in the Cipro group and 986 in the CiproAMG group (Table 1). Ciprofloxacin was augmented with tobramycin and gentamicin in 920 (93.3%) and 66 (6.7%) biopsies, respectively. From 2010 to 2018, the administration of ciprofloxacin alone followed a significant negative trend (*p* = 0.004), whereas the administration of the augmented regimen followed a significant positive trend (*p* < 0.001). Figure 1 shows the amount of TRUSPBs that were performed between 2010 and 2018 and the prophylaxis regimen that was given. The total amount of TRUSPBs decreased from 2010 to 2014 but increased as of 2015.

**Figure 2:**
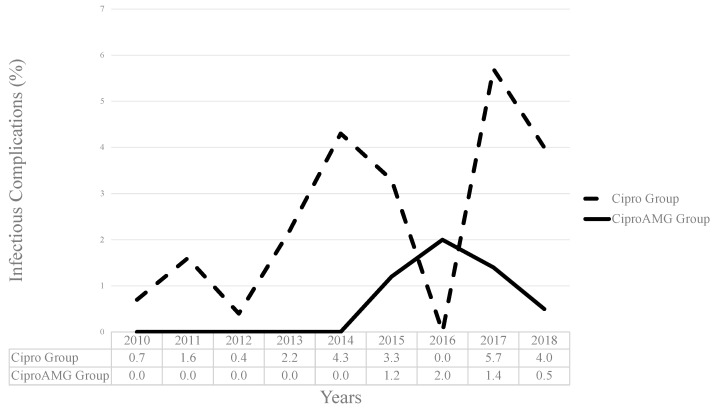
Pooled post-prostate biopsy infectious complications rates from 2010 to 2018. The rates per year are shown in a linear trend and are stratified according to groups (Cipro group and CiproAMG group).

**Figure 3:**
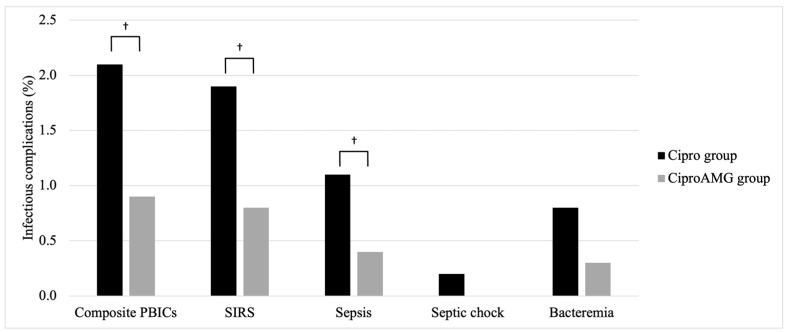
Individual and pooled post-prostate biopsy infectious complications rates from 2010 to 2018. The bar graph represents the rates per post-individual infectious complication and is stratified according to groups (Cipro group and CiproAMG group). ^†^ *p* < 0.05.

Between 2010 and 2018, the PBIC rates in the two groups significantly differed: 2.1% (*n* = 39) in the Cipro group, and 0.9% (*n* = 9) in the CiproAMG group (*p* = 0.019; OR, 0.43; 95% confidence interval [CI], 0.21–0.89). Figure 2 shows the evolution of PBIC rates, and Figure 3 depicts the composite and individual PBIC rates according to antibiotic prophylaxis received. The CiproAMG SIRS and sepsis rate was significantly lower than that of the Cipro group (0.8% vs. 1.9% for SIRS and 0.4% vs. 1.1% for sepsis) (*p* = 0.020; OR, 0.41; 95% CI, 0.19–0.89 for SIRS and *p* = 0.048; OR, 0.36; 95% CI, 0.12–1.04 for sepsis). A sufficient sample size was not obtained to establish a statistical difference between the two groups regarding bacteremia or septic shock. The composite PBIC rate from the 2010–2013 period to the 2014–2018 period was significantly different in the Cipro group (1.6% to 3.7%, *p* = 0.006) but not in the CiproAMG group (0% to 1.1%, *p* = 0.202). No PBICs were detected in the CiproAMG group from 2011 to 2013. The overall PBIC rate was still significantly higher in the Cipro group during the 2014–2018 period (*p* = 0.001; OR, 0.28; 95% CI, 0.13–0.64).

Factors such as age, the number of cores removed, having a TRUSPB and a UTI treated within the last year, or following a FQ regimen 6 months prior to the biopsy significantly differed between the Cipro and CiproAMG group (Table 1).

The augmented regimen was associated with a significantly lower PBIC rate, independent of risk factors (*p* = 0.014; OR, 0.40; 95% CI, 0.19–0.83). Taking a FQ in the previous 6 months (*p* = 0.009) and having a UTI 1 year prior to the biopsy (*p* = 0.011) were shown to have a significant impact on PBIC rates with the univariate analysis (Table 2). Hospitalization and UTI within the previous year and FQ use within the previous 6 months were all included in the multivariate analysis but failed to significantly impact PBIC rates. A secondary multivariate analysis was performed using these three factors incorporated into a single composite variable and the treatment as independent variables. The newly composite variable was shown to significantly impact the composite PBICs’ rate (*p* = 0.039; OR, 2.51; 95% CI, 1.05–6.00).

A total of 26/39 (66.7%) patients with a PIBC in the Cipro group and 6/9 (66.7%) patients with a PIBC in the CiproAMG group were hospitalized within 30 days post-biopsy. Average length of stay was 6.6 days and 6.7 days, respectively. No significant differences were found between the groups regarding post-biopsy hospitalization. No mortality was reported.

Out of the 48 patients with a PBIC, 29 (60.4%) had positive *E. coli* urine and/or blood culture. No other pathogens were identified in cultures. A total of 5 *E. coli* were in the CiproAMG group, and 24 were in the Cipro group. Average time of culture after the biopsy was 3.0 days. A total of 28 *E. coli* species (96.6%) were found to be ciprofloxacin-resistant: 24 (100%) in the Cipro group, and 4 (80.0%) in the CiproAMG group. In the latter, two *E. coli* species were AMG-resistant, while three were AMG-sensitive. In the Cipro group, 24 cases of SIRS (66.7%) and 17 cases of sepsis (81.0%) presented with AMG-sensitive strains.

## 4. Discussion

In our study, the augmented prophylaxis consisting of an 80 mg aminoglycoside dose added to the standard ciprofloxacin prophylactic regimen significantly reduced the composite PBIC rate compared to the use of ciprofloxacin alone. The infection rate in the Cipro group (2.1%) was more than two-fold lower than what is generally reported (approximately 5%) [3]. At the end of the study, the composite PBIC rate (i.e., SIRS, sepsis, septic shock, and bacteremia) was significantly lower in the CiproAMG group compared with that of the Cipro group (0.9% vs. 2.1%). Impacting a composite endpoint of infectious complications is associated with a significant reduction in hospital length of stay, cost, and mortality [20].

SIRS and sepsis were the only complications, when assessed separately, that had a significantly lower rate in the CiproAMG group. The augmented regimen did not significantly affect other separately measured complications (i.e., septic shock and bacteremia), although lower rates were reported in the CiproAMG group for each of the complications (Figure 3). No cases of septic shock were reported with the augmented regimen. Adding an AMG to a FQ-based prophylactic regimen has previously been shown to significantly decrease the rate of bacteremia [22]. The impact of decreasing the incidence of a single complication on patient outcomes in this population is undetermined. However, SIRS increases the rate of in-hospital death by almost four-fold [26], while sepsis overall is associated with an in-hospital mortality exceeding 10%, and septic shock is associated with an in-hospital mortality rate exceeding 40% [24].

Based on the rapidly increasing PBIC rates [1,2,3] concomitant to rising FQ-R *E. coli* occurrences [2,5,6], the use of fluoroquinolones in empirical prophylaxis is being questioned. Targeted prophylaxis as an alternative is not always successful at reducing PBIC [15,16,17,18]. This is possibly due to suboptimal detection sensitivity and/or errors in rectal flora sampling. Augmented prophylaxis with the addition of an AMG to a ciprofloxacin regimen is demonstrated to impact PBIC rates [20,21,22,27]. This large cohort is confirmed by recent findings that ciprofloxacin augmented with a single dose of gentamicin significantly reduces PBIC rates when compared with standard or targeted prophylaxis [21]. Ciprofloxacin augmented with fosfomycin tromethamine could be an adequate alternative if confirmed in a randomized trial [28].

Gentamicin and tobramycin were administered as they are both effective in decreasing PBIC rates while being cost-effective [20,27]. Our results confirm these previous observations. Supplementing the antibiotic prophylaxis regimen with an AMG broadens the spectrum of activity to cover resistant bacteria. However, the findings here of pathogens susceptible to the augmented regimen may be of concern for their ability to fully prevent gram-negative bacteria-associated complications. Of the five *E. coli*-positive patients in the CiproAMG group, three (60%) had an AMG-sensitive strain. These three patients developed a PBIC despite receiving both ciprofloxacin and an aminoglycoside. Dosing could affect the efficacy of AMGs. The addition of gentamicin at 240 mg significantly decreased infectious complications, while a dose of 80 mg or 120 mg failed to produce a significant impact in Israel [29]. In our study, an intramuscular dose of 80 mg was used for practical reasons and was sufficient to be associated with decreased PBIC rates.

The significant decrease of PBICs in the CiproAMG group was found to be an independent risk factor. When performing a prostate biopsy, repeated inoculation may increase the risk of introducing bacteria from the rectal flora. Removing 12 cores is considered standard; however, removing more did not have a significant impact on complications rate, as previously reported [30]. Prior hospitalization increases the risk of exposure to resistant bacterial strains but was not shown to be a significant risk factor in this study. Depending on local resistance patterns, some hospitals may be at greater risk than others. Undergoing a TRUSPB prior to the one assessed in the study increased the chances of harboring FQ-R organisms but failed to significantly impact PBIC rates. Taking a single dose of ciprofloxacin increased the risk of developing resistance by four-fold [31]. As expected, having followed a prior FQ regimen significantly impacted PBIC rates. In line with this rationale, a prior UTI also had a significant impact on PBIC rates. Despite efforts to discontinue ciprofloxacin as a first-line agent when treating UTIs, the latter is still frequently used. Being exposed to a FQ regimen 6 months prior to the biopsy, having a UTI 1 year before the biopsy, and being hospitalized within the previous year are all significant in the multivariate analysis as composite endpoints.

Although the mechanism behind a PBIC is not well-understood, it is believed that *E. coli* from the rectal flora gains access to the bladder and/or bloodstream following the insertion of the needle, resulting in bacteremia. The culture results for *Enterobacterales* spp. are similar those found in the literature [7,8]. Most infections were caused by *E. coli* (58%), as previously reported [2,5,6,7,8]. No pathogens were identified in the remaining group. Following the administration of ciprofloxacin alone, more than 95% of *E. coli* strains were found to be FQ-R in patients developing a PBIC, confirming previous observations [6,31]. When receiving a FQ alone, the presence of resistant bacteria in the rectal flora significantly increased PBIC rates by four-fold [10]. These findings, combined with our results, confirm that augmented prophylaxis is a favorable alternative to the currently recommended empirical prophylaxis regimen that is failing.

The limitations of this retrospective study should be considered. The lack of documentation regarding prophylaxis resulted in the elimination of large number of patients. The quality of surgical procedures can improve or change over time, according to surgeons’ experience and impact outcomes. The retrospective nature of this study did not allow the recollection of all the desired risk factors. The number of patients in each risk factor group is a limitation to the interdependent relationship of risks. Having a higher rate of risk factors that significantly impact PBIC occurrence (i.e., UTI < 1 year and FQ use < 6 months) in the CiproAMG group may result in an overvaluation of PBIC rate in this group. However, this potential bias was considered in the multivariate analysis. Previous antibiotic exposure could only be tracked for antibiotics prescribed by MUHC physicians or administered at the MUHC. The impact of outpatient exposure to antibiotics could not be assessed. The impact of other practices not implemented here, such as screening for fluoroquinolone resistance to adapt antibiotic choice before the procedure, were not assessed. Ciprofloxacin-tobramycin *E. coli* resistance rates remained low in our cohort. Local patterns with higher rates may preclude the use of a Cipro-AMG regimen. Nevertheless, our study confirms that the use of augmented prophylaxis with an aminoglycoside leads to significantly lower infectious complications following a transrectal ultrasound-guided prostate biopsy.

## 5. Conclusions

In summary, an augmented prophylaxis regimen consisting of a single intramuscular 80 mg AMG dose (gentamicin or tobramycin) and ciprofloxacin is more effective in preventing PBIC than ciprofloxacin alone. The results of this study support the use of augmented prophylaxis with the aim of covering for FQ-R bacteria, among others. Breakthrough infectious complications with susceptible strains to aminoglycosides require further investigation. This study illustrates the impact of augmented prophylaxis on PBIC rates compared with that of empirical prophylaxis in a population with a low incidence of infections.

## Figures and Tables

**Table 1:** Patients’ characteristics stratified by treatment group.

Population Characteristics	Overall (*n* = 2835)	Cipro Group (*n* = 1849)	CiproAMG Group (*n* = 986)	*p*-Value ^a^
**Demographics**				
Age, years (mean [SD])	67.0 (8.7)	66.6 (8.8)	67.8 (8.5)	<0.001
Weight ^b^, kg (mean [SD])	80.1 (16.8)	79.0 (18.0)	81.9 (14.6)	0.121
**Biopsy**				
Year (*n* [%])				
2010	406 (14.3%)	406 (22.0%)	0 (0%)	
2011	450 (15.9%)	368 (19.9%)	82 (8.3%)	
2012	382 (13.5%)	337 (18.2%)	45 (4.6%)	
2013	301 (10.6%)	278 (15.0%)	23 (2.3%)	
2014	179 (6.3%)	92 (5.0%)	87 (8.8%)	
2015	223 (7.9%)	62 (3.4%)	161 (16.3%)	
2016	265 (9.3%)	69 (3.7%)	196 (19.9%)	
2017	287 (10.1%)	86 (4.7%)	201 (20.4%)	
2018	342 (12.1%)	151 (8.2%)	191 (19.4%)	
Number of cores removed (mean [SD])	11.4 (1.6)	10.9 (1.6)	12.3 (1.1)	<0.001
**Risk factors**				
Number of cores > 12 (*n* [%])	503 (17.7%)	296 (26.0%)	207 (21.0%)	0.001
TRUSPB < 1 year (*n* [%])	202 (7.1%)	149 (8.1%)	53 (5.5%)	0.008
Hospitalization < 1 year (*n* [%])	135 (4.8%)	85 (4.6%)	50 (5.1%)	0.573
UTI treated < 1 year (*n* [%])	41 (1.4%)	17 (0.9%)	24 (2.4%)	0.001
FQ use < 6 months (*n* [%])	65 (2.3%)	33 (1.8%)	32 (3.2%)	0.013

^a^ A Student’s *t*-test was used to compare continuous variables; using Levene’s test, equality of variance was assumed for weight. A Pearson’s Chi-square test was used to compare categorical variables. ^b^ Weight was documented in 211 patients in the Cipro group and in 133 patients in the CiproAMG group. Abbreviations: TRUSPB, transrectal ultrasound-guided prostate biopsy; SD, standard deviation; UTI, urinary tract infection; FQ, fluoroquinolone.

**Table 2:** Univariate and multivariate logistic regression analysis for association with infectious complications.

	Univariate Analysis		Multivariate Analysis ^a^	
	OR (95% CI)	*p*-Value	OR (95% CI)	*p*-Value
Treatment	0.43 (0.21–0.89)	0.022	0.40 (0.19–0.83)	0.014
Age	1.00 (0.96–1.03)	0.803		
Number of cores > 12	1.23 (0.61–2.47)	0.572		
TRUSPB < 1 year	1.53 (0.60–3.90)	0.376		
Hospitalization < 1 year	2.38 (0.93–6.10)	0.072	1.35 (0.41–4.47)	0.661
FQ use < 6 months	4.06 (1.42–11.66)	0.009	2.32 (0.42–12.71)	0.333
UTI treated < 1 year	4.82 (1.44–16.20)	0.011	2.48 (0.39–15.80)	0.336

^a^ Only risk factors obtaining a *p*-value below 0.1 using the univariate analysis were analyzed using the multivariate analysis. Abbreviations: OR, odds ratio; CI, confidence interval; TRUSPB, transrectal ultrasound-guided prostate biopsy; FQ, fluoroquinolone; UTI, urinary tract infection.

## Data Availability

Not applicable.
